# The Exit of Mouse Embryonic Fibroblasts from the Cell-Cycle Changes the Nature of Solvent Exposure of the 5′-Methylcytosine Epitope within Chromatin

**DOI:** 10.1371/journal.pone.0092523

**Published:** 2014-04-04

**Authors:** Selcen Çelik, Yan Li, Chris O'Neill

**Affiliations:** Human Reproduction and Development Unit, Kolling Institute for Medical Research, Sydney Medical School, University of Sydney, Sydney, Australia; Université Paris-Diderot, France

## Abstract

The methylation of CpG dinucleotides is a pervasive epigenetic signature with critical roles governing genomic stability and lineage-specific patterns of gene expression. Reprogramming the patterns of CpG methylation accompanies key developmental transitions and the onset of some pathologies, such as cancer. In this study we show that levels of immuno-detectable 5meC decreased as mouse embryonic fibroblasts withdraw from the cell-cycle (became mitotically quiescent), but increased as they aged in culture. Two pools of 5meC epitope were found to exist, one solvent exposed after acid-induced denaturation of chromatin and another that required the additional step of tryptic digestion for detection. Proliferative cells displayed a relatively greater accumulation of detectable 5meC within the trypsin-sensitive pool than did quiescent cells. A substantial proportion of the 5meC was associated with a large number of heterochromatic foci scattered throughout nuclei, yet much of this was masked in a trypsin-sensitive manner, particularly in young proliferative cells. This study showed that the growth status of cells changed the level of solvent exposure of 5meC in fibroblasts and the long-accepted conventional methods of immunolocalization underestimate the level of 5meC in cells. This resulted in an artefactual assessment of the levels and patterns of nuclear localization of the antigen. The use of an additional tryptic digestion step improved antigen retrieval and revealed a more dynamic response of 5meC levels and distribution patterns to changes in the cell's growth state. This discovery will provide a basis for investigating the role of changes in chromatin structure that underlie this dynamism.

## Introduction

Lineage specific patterns of CpG methylation (5-methylcytosine, 5meC) are considered to make an important contribution to the cell's epigenetic landscape [1], [2]. The extent of methylation of regions of the genome influences patterns of gene expression and has an important role in the maintenance of genomic stability [3]. Large changes in the levels of methylation have been reported to accompany important biological transitions. These include reports of genome-wide loss of methylation in the early embryo immediately following fertilisation and during the formation of primordial germ cells [4]. It is generally considered that these reported changes contribute to the process of epigenetic reprogramming towards the pluripotent state of these cells.

It has long been held (based primarily upon immunolocalization analyses) that a round of global active demethylation of the paternally-inherited genome occurs relative to the maternally-inherited genome soon after fertilisation [5]–[7]. Recent chemical analyses, however, showed relatively similar overall levels of methylation in both the male and female pronucleus of the zygote [8] and persistence of methylation levels in blastocysts [9], rather than the marked relative hypomethylation of the paternally-derived genome indicated by immunolocalization studies [10]. Detailed re-analysis of the immunostaining methodology in the zygote showed that reports of active loss of methylation from the paternal genome were a manifestation of a progressive onset of acid-resistant masking of the 5meC antigen rather than its loss [11], [12]. Brief digestion of the fixed cells with trypsin revealed that the levels of 5meC were in fact maintained during this period of zygotic maturation. It was concluded that changes in chromatin organisation during the zygotic maturation resulted in a progressive trypsin-sensitive reduction in the solvent exposure of the 5meC epitope even after acid-induced denaturation of chromatin. The nature of these changes to chromatin is yet to be defined.

Since immunolocalization of 5meC is an important tool in the analysis of this epigenetic feature, it is of interest to understand whether the masking of the antigen observed during zygotic maturation is unique to the zygote or whether it more generally affects the ability to reliability detect levels and localization of 5meC within cells. A notable feature of the zygote is that it is a key developmental transition whereby cells that have left the cell-cycle (the gametes, sperm and oocyte) become mitotically active again upon fertilisation. Somatic cells can also reversibly leave the cell-cycle to enter a mitotically quiescent phase and upon appropriate activation re-enter the cell-cycle. This phenomenon has been studied extensively in primary fibroblast in vitro. A range of conditions such as the withdrawal of growth factors or extensive confluence of cells in culture induces the exit of cells from the active cell-cycle by entering the G_0_ phase. This phase is characterised a reversible mitotic quiescence, and is accompanied by changes in the patterns of gene expression and chromatin organisation [13], [14]. Removal of these conditions (for example, by creating sparse cultures in the presence of growth factors) allows cells to pass a ‘restriction point’ and re-enter the cell-cycle at G1.

In this study we examined the effects of entry of mouse primary fibroblast into quiescence on the levels and localization of 5meC detected by conventional immunolocalization, and the effect of this change in the growth status on the relative levels of trypsin-sensitive masking of 5meC. The total immuno-detectable pool of 5meC was higher in proliferative cells than quiescent cells, but the 5meC existed in two pools; one detected after acid-induced denaturation of chromatin, and another pool that required a further step of tryptic digestion. The trypsin-sensitive pool of 5meC was a larger proportion of the total staining in proliferative than quiescent cells. This finding has important implications for the interpretation of immunolocalization analysis of cellular 5meC. It reinforces the need to fully validate the immunolocalization methodology for each cell type and model under analysis. The finding of changes in solvent exposure of 5meC in two different models of biological transitions indicates that this may be a biologically relevant feature of the epigenetic landscape that requires more extensive investigation.

## Results

**Figure 1:**
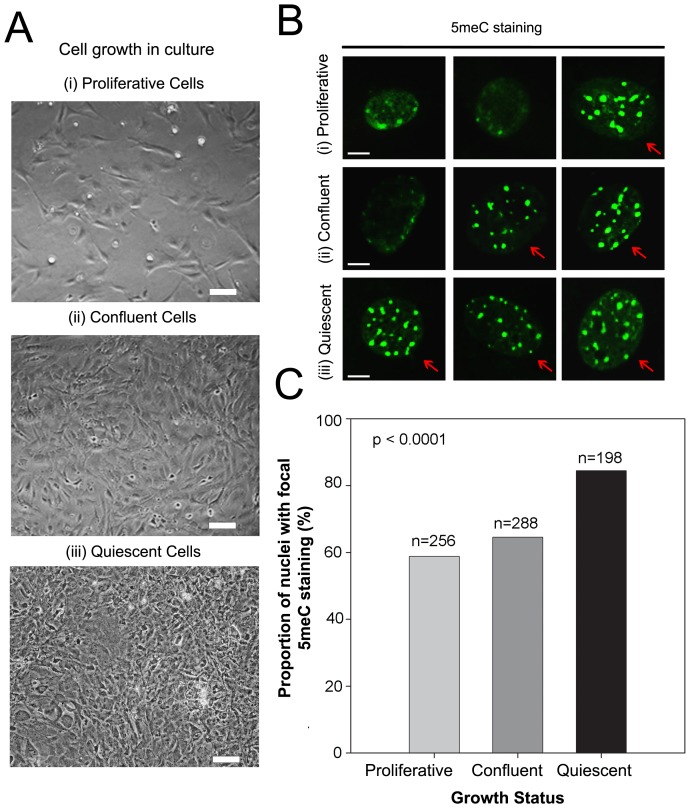
The staining pattern of 5meC within nuclei in MEFs. **A** Phase contrast images of the growth pattern of MEFs in (i) sparse (proliferative), (ii) confluent, and (iii) serum deprived confluent (quiescent) cells. Bar = 20 µm. **B** Typical examples of the 5meC staining patterns of MEFs in (i) proliferative, (ii) confluent, and (iii) quiescent growth phases. Arrows show cells with a predominantly focal pattern of staining and the other cells show a predominantly diffuse pattern of staining. Scale bar 5 µm. **C** The proportion (%) of nuclei with predominantly focal 5meC staining in each cell (n, number of nuclei analysed). The focal pattern of 5meC increased with cell proliferation (p<0.0001). The results are representative of three independent replicates. Epitope retrieval was by treatment with of cell with HCl 4N for 10 min at 22°C.

The localization of 5meC within the nucleus was assessed by examining cells in three different growth states: (i) during the exponential growth phase of sparse culture where most cells are undergoing proliferation (proliferative); (ii) as proliferation slowed due to approaching confluence of cells (confluent); and (iii) as cells enter into quiescence by serum deprivation for 24 h of confluent cells (quiescent) (**Fig. 1A**). The distribution of 5meC staining using conventional immunostaining methodology (with epitope retrieval by brief acid treatment) differed between treatments. Subjectively, cells were classified as showing primarily either a diffuse or focal pattern of 5meC staining. Diffuse staining was characterised by a pattern of relatively low intensity 5meC staining across the nucleoplasm, with only a small number of areas of more intense staining (**Fig. 1B**). Focal staining was characterised by a pattern whereby a large number of distinct 5meC-rich foci were observed scattered throughout the nuclei (focal staining represented by arrows- **Fig. 1B**). Using this subjective classification, differences in the incidence of each pattern of staining occurred with the growth status of the cells. A higher proportion of cells (p<0.0001) was found to have a predominantly focal pattern of staining (**Fig. 1C**) as they left the cell-cycle by becoming confluent and then quiescent (by serum withdrawal).

**Figure 2:**
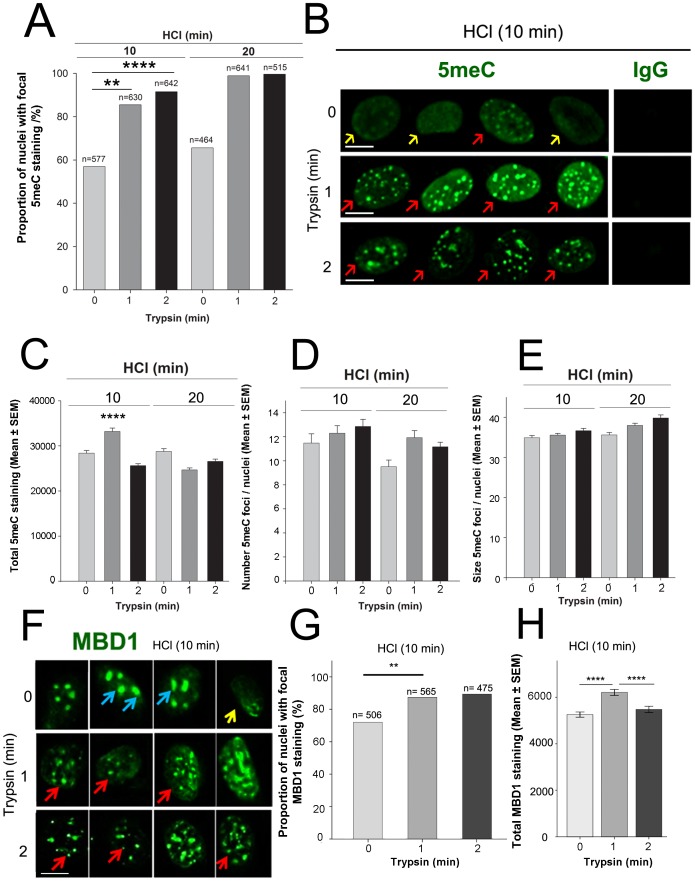
Staining pattern of 5meC and MBD1 antigens after epitope retrieval acid or acid plus trypsin treatment of confluent MEFs. Fixed and permeabilized MEFs were treated with HCl for 10 or 20**A** The proportion of nuclei (%) with focal 5meC staining (n, number of nuclei analysed). HCl (p<0.01) and trypsin (p<0.01) treatments revealed an increase in the focal staining of nuclear 5meC. 20 min HCl caused no further increase (p>0.05) after trypsin. **B** Typical examples of focal and diffuse staining of 5meC staining within the nuclei, yellow and red arrows indicate predominantly diffuse and focal staining, respectively. Non-immune IgG control staining is also shown. **C** The total level of 5meC staining of treatments shown in A. The results are the mean ± SEM of arbitrary units of optical density increased (**** p<0.0001). **D** The average number of 5meC foci per cell in A. **E** The average size (cross-sectional area) of 5meC foci in cells in A. **F** Representative images of MBD1 staining after 10 min HCl and trypsin 0, 1 or 2 min. Blue and red arrows indicate large and small foci of MBD1, respectively, and yellow arrow shows diffuse staining of MBD1. **G** The proportion of nuclei with focal MBD1 staining increased after 1 min trypsin (p<0.01). **H** The total level of MBD1 staining (arbitrary units of optical density) increased after 10 min HCl and 1 min trypsin (p<0.0001) Mean +/− standard mean of error. Results are from three independent replicates. ** p<0.01 and **** p<0.0001. Scale bar 10 µm. If not shown on individual graphs, the relevant statistical outcomes are stated within the results text.

To determine whether the method of antigen retrieval influenced the detection of 5meC staining, we performed a factorially-designed experiment to assess the relative effects of acid-induced denaturation of chromatin compared to epitope retrieval by acid-treatment followed by tryptic-digestion. In the absence of either HCl or trypsin treatment little detectable 5meC staining was observed in proliferative cells (not shown). After 10 or 20 min treatment with 4 N HCl, a similar proportion of cells showed a focal pattern of staining, but the proportion of cells with focal staining (p<0.01) (represented by red arrows **Fig. 2A, 2B**) and the level of total 5meC staining (**Fig. 2B, 2C**) increased (p<0.0001) after 10 min HCl and an additional 1 min digestion with trypsin (0.25%). There was no additive benefit of 20 min HCl when acid treatment was followed by tryptic digestion (p>0.05) (**Fig. 2A**). There was no overall effect of trypsin treatment or HCl treatment on the total 5meC staining level, but 10 min of acid treatment with 1 min trypsin did reveal higher levels of staining than other treatments (**Fig. 2C**). The number of 5meC-rich foci detected per cell also increased (p<0.05) after tryptic digestion (but not acid treatment) (**Fig. 2D**), as did the average size (cross-sectional area) of each 5meC focus (p<0.001) (**Fig. 2E**). The staining pattern of MBD1 (a putative proxy measure of 5meC) was tested after acid and trypsin treatment. After HCl treatment alone (10 min), MBD1 staining was observed within the large nuclear foci (blue arrows **Fig. 2F**) while a low number of nuclei showed diffuse staining of MBD1 (yellow arrow **Fig. 2F, 2G**). HCl followed by 1 min trypsin resulted in a large number of smaller nuclear foci of MBD1 staining (red arrows **Fig. 2F**). The proportion of nuclei with focal MBD1 staining (p<0.01) (**Fig. 2F, 2G**) and the level of detectable MBD1 staining (p<0.0001) (**Fig. 2F, 2H**) also increased after trypsin for 1 min. Based on these findings a standard condition for both 5meC and MBD1 epitope retrieval of 10 min HCl at 22°C followed by 1 min trypsin at 37°C was used in subsequent studies unless otherwise stated.

**Figure 3:**
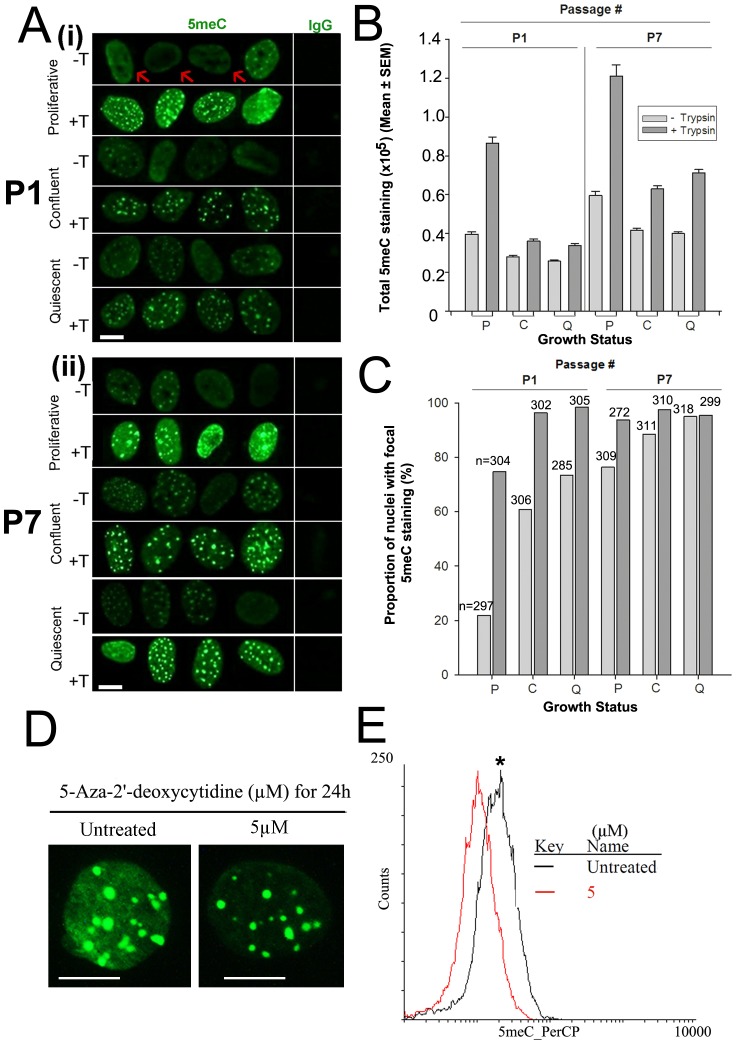
The effects of growth status on the pattern of cytosine methylation. **A** Proliferative, confluent and quiescent cells at (i) passage 1 (P1) or (ii) passage 7 (P7) were stained with 5meC after 10 min acid (−T) or acid treatment followed by 1 min trypsin (+T). Scale bar 10 micron. Typical diffuse 5meC staining is shown by red arrows. **B** The total level of 5meC staining. Proliferative (P); Confluent (C); quiescent (Q). Growth status (p<0.0001) affected both the level of 5meC detected and the proportion of nuclei with focal 5meC and the use of trypsin (p<0.0001) affected the detection of those. **C** The proportion (%) of nuclei with focal 5meC staining. n, total nuclei analysed. Passage # (p<0.0001) affected both the level of 5meC detected and the proportion of nuclei with focal 5meC and the use of trypsin (p<0.0001) affected the detection of those. The results are from three independent replicates. **D** The effect of treatment of proliferative cells for 24 h with the 5 µM 5-aza-2′-deoxycytidine-2′-deoxycytidine on the level of 5meC staining of MEFs. Scale bar = 10 µm **E** The decrease in the staining levels of 5meC in MEFs after treatment with 5 µM 5-aza-2′-deoxycytidine-2′-deoxycytidine compared to control cells (Untreated) as detected by fluorescence activated flow cytometry (p<0.05). 5meC is tagged with a PerCP-conjugated antibody and detected at fluorescence channel-3. * p<0.05.

The level of acid- and trypsin-sensitive masking of total and focal 5meC staining in proliferative, confluent and quiescent cells was assessed in young (Passage 1, P1) and older (P7) cells (**Fig. 3**). In both groups there was more total acid-sensitive 5meC staining detected in proliferative cells than those withdrawing from the cell-cycle (p<0.001) (**Fig. 3A, 3B**). Overall, older cells (P7) had more staining than younger cells (P1) (p<0.001). Tryptic digestion caused more total nuclear 5meC staining to be detected in all treatments (p<0.001), and this differential was greatest in proliferative cells (p<0.001) (**Fig. 3B**). The magnitude of change in the level of staining detected after tryptic digestion was greater than the effects caused by the proliferative status or age of cells in culture, but the magnitude of the difference in staining detected in proliferative compared to quiescent cells was much greater after tryptic digestion (**Fig. 3B**). Fewer proliferative cells displayed a predominantly focal pattern of staining compared to confluent and quiescent cells (p<0.001) (**Fig. 3C**). This effect was greater in young cells (P1) in the absence of tryptic digestion (p<0.0001) (**Fig. 3A, 3C**). After tryptic digestion the increase in 5meC staining occurred across the entire nucleoplasm including areas that showed focal patterns of staining. Tryptic digestion caused an increase in the proportion of cells with focal staining across all treatment groups (p<0.0001) (**Fig. 3C**). This relative increase was greatest in younger cells (due to the low levels of 5meC detected in cells not subjected to trypsin treatment), and this differential was greater in both young and old proliferative cells. This result shows that the proliferative status and age of cells had an effect on the level of both acid-sensitive and trypsin-sensitive solvent exposure of the 5meC epitope. These treatments also had an impact on the detection of the focal distribution of 5meC within cells, and this was most apparent in younger, proliferative cells. Tryptic-digestion had the effect of increasing the total levels of 5meC detected across the nucleoplasm and revealed a higher incidence of focal 5meC staining patterns in most treatments than was evident after acid-induced denaturation alone.

To assess the capacity of this methodology to detect global changes in total levels of 5meC, we next treated proliferative cells with the DNA methyltransferase inhibitor, 5-aza-2′-deoxycytidine. This drug inhibits maintenance methylation of the newly replicated DNA strand during the cell-cycle [15]. Proliferative cells were cultured for 24 h in the presence of 0 or 5 µM 5-aza-2′-deoxycytidine. Immunolocalization showed that a clear reduction in the level of 5meC after 5-aza-2′-deoxycytidine treatment (**Fig. 3D**). The reduction in staining was most evident in the regions of the nucleoplasm outside the 5meC staining-intense foci. Analysis was then repeated using fluorescence-activated cytometry to provide a more quantitative measure on a large number of cells. This also showed a reduction in the mean level of 5meC detected after 5-aza-2′-deoxycytidine treatment (p<0.05) (**Fig. 3E**). Immunolocalization of 5meC after epitope retrieval by acid and trypsin treatment allows for the detection of global changes in the level of 5meC within MEFs. Immunolocalization also has the advantage of allowing visualisation of changes in the distribution of staining within individual nuclei.

**Figure 4:**
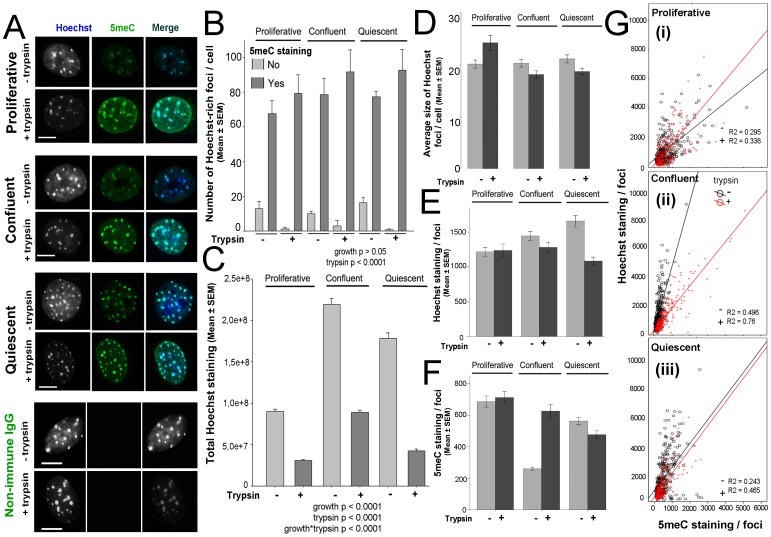
The effect of cell growth on the pattern of co-staining for 5meC and bisBenzimide (Hoechst). **A** co-staining of 5meC and DNA by Hoechst in cells during proliferation after acid alone (−trypsin) or acid followed by trypsin (+trypsin). 5meC foci are co-localized with Hoechst-dense regions in each cell. Representative non-immune IgG staining in proliferative cells is also shown. Scale bar 10 µm. **B** The number of Hoechst-dense foci per cell co-stained with 5meC or failed to co-stain with trypsin (+) or without (−). Tryptic digestion significantly (p<0.0001) affected the detection of co-localization of 5meC and DNA-dense areas. **C** The level of Hoechst staining (arbitrary units of optical density) of cells with trypsin (+) or without (−) during proliferation. Trypsin revealed a decrease in the detection of amount of Hoechst staining in each cell (p<0.0001) and cell growth affected Hoechst staining (p<0.0001). **D** Average size of Hoechst-rich foci per cell (arbitrary units of optical density). **E** The level of Hoechst foci staining per cell. **F** The level of 5meC foci staining per cell. Trypsin decreased the detectable amount of Hoechst and 5meC foci (p<0.0001). Cell growth affected the 5meC (p<0.0001) but not Hoechst (p>0.05). **G** The relationship between Hoechst and 5meC foci staining for each nucleus in (i) proliferative, (ii) confluent and (iii) quiescent cells (p<0.0001). Hoechst and 5meC are positively correlated in each growth. R-squared for each growth is given. The results are of three independent replicates.

BisBenzimide (Hoechst stain) accumulates in heterochromatic regions of the nucleus and its staining showed a pattern of extensive focal distribution of presumptive heterochromatin scattered throughout the nuclei in each of the three growth states (**Fig. 4A**). There were a similar total number of these foci irrespective of the cell's proliferative status (p>0.05) but the number detected showed a small increase after tryptic digestion (p>0.001) (**Fig. 4A, 4B**). The total level of Hoechst staining was affected by both the cell's growth status (p<0.0001) and tryptic digestion (p<0.0001), with tryptic digestion causing a marked overall reduction in the level of total Hoechst staining in nuclei under all growth conditions (**Fig. 4C**). This occurred despite the small increase in the number of foci (**Fig. 4D**) caused by tryptic digestion, but was associated with a reduction in the average level of the Hoechst staining in each staining focus of confluent and quiescent cell (but not proliferative cells) (**Fig. 4E**). This result shows that trypsin reduced Hoechst staining levels across the nucleoplasm, including within the staining intense foci of cells leaving the cell-cycle. Co-staining of cells for 5meC and Hoechst showed a high degree of co-incident staining for 5meC and Hoechst in each growth state (**Fig. 4A**). This coincident staining was higher after acid-only epitope retrieval compared to those subjected to tryptic digestion (**Fig. 4F**). Overall the average level of 5meC staining in individual foci declined as cells withdrew from the cell-cycle and this trend was most evident in cells subjected to tryptic digestion. In confluent cells the 5meC staining level in individual foci was lower than in any other treatment (**Fig. 4F**). Analysis of the 5meC and Hoechst staining in individual foci showed that there was an overall strong positive association between these two measures (**Fig. 4G**). In proliferative (**Fig. 4G-i**) and quiescent (**Fig. 4G-iii**) cells this relationship was similar with or without tryptic digestion (p>0.05), while for confluent cells (**Fig. 4G-ii**) trypsin treatment caused more 5meC to be detected relative to Hoechst (p<0.01). The results show that the changes in chromatin structure caused by brief tryptic digestion reduced the amount of Hoechst bound and were associated with increased exposure of the 5meC epitope. This effect varied, however, with the growth status of fibroblasts. This indicates that the heterochromatic state of chromatin may be a determinant of the solvent exposure of 5meC, and one consequence of tryptic digestion may be to relieve this heterochromatic state.

**Figure 5:**
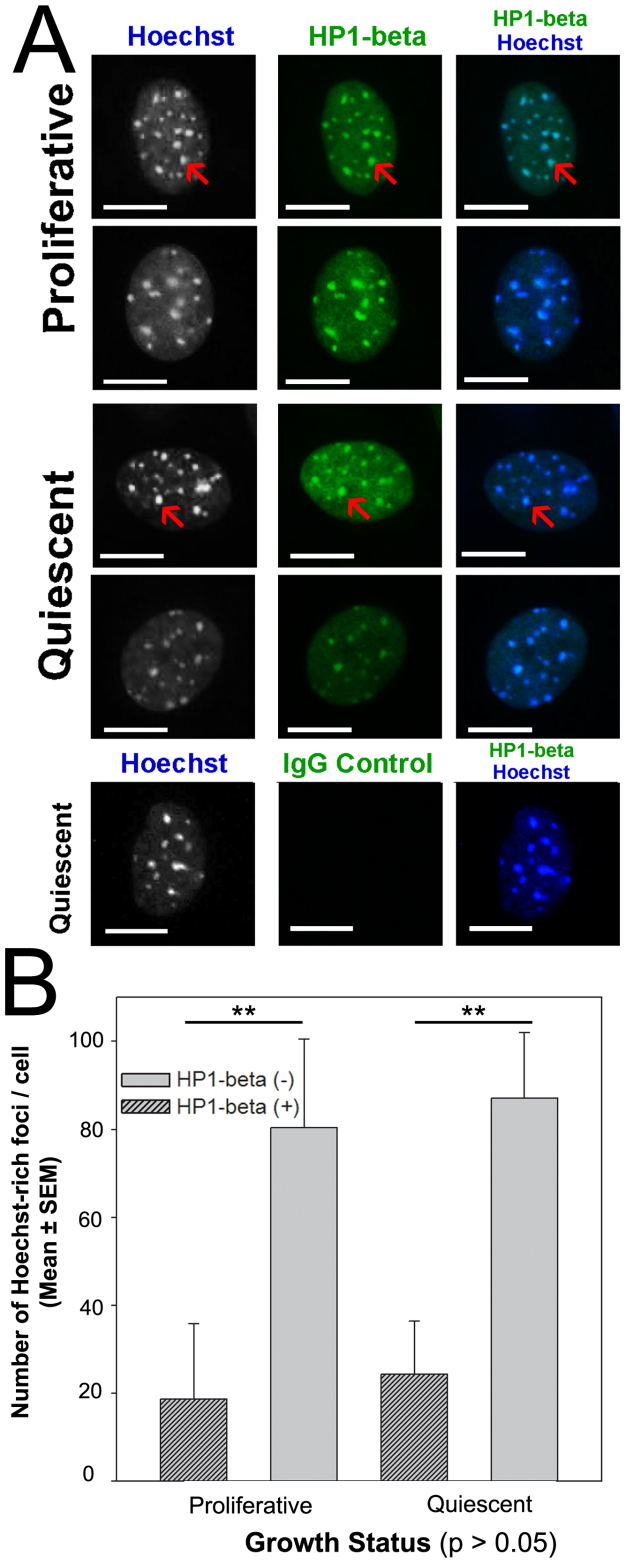
The effect of cell growth on the pattern of co-staining for heterochromatin (HP1-β) and bisBenzimide (Hoechst 33342). **A** co-staining of HP1-β (and non-immune IgG control) and Hoechst in proliferative and quiescent cells. Scale bar 10 micron. **B** The number of Hoechst-dense foci co-stained with HP1-β (+) or not (−). HP1-β co-localized with Hoechst foci (p<0.01) irrespective of cell proliferation (p>0.05). Mean + i.e. The results are of three independent replicates. ** p<0.01.

The HP1β protein is widely used as a specific marker of heterochromatin [16]–[18] so we next examined whether the Hoechst-intense foci coincided with HP1-β staining in proliferative and quiescent cells (**Fig. 5**). We found that staining for this protein was lost after tryptic digestion (not shown). Cells were therefore assessed after acid-based antigen retrieval only. **Fig. 5A** shows that there was a low level of HP1β staining throughout the nucleoplasm of cells in each treatment, and this was accompanied by a number of intense-focal regions of staining. Most of these HP1β-intense foci coincided with Hoechst foci (represented by red arrows, **Fig. 5A**), although around 20% of the Hoechst foci in both proliferative and quiescent cells did not show an accumulation of HP1β staining. Heterochromatin therefore seemed to possess similar general staining features in both proliferative and quiescent cells. Approximately 25% of the Hoechst-intense foci in proliferative cells were not decorated by anti-HP1β (derived from **Fig. 5B**), which is similar to the proportion of Hoechst-intense foci that were not decorated by anti-5meC (**Fig. 4B**). The methodology did not allow us to assess directly whether the Hoechst-intense foci that lacked HP1β and 5meC were the same pool.

**Figure 6:**
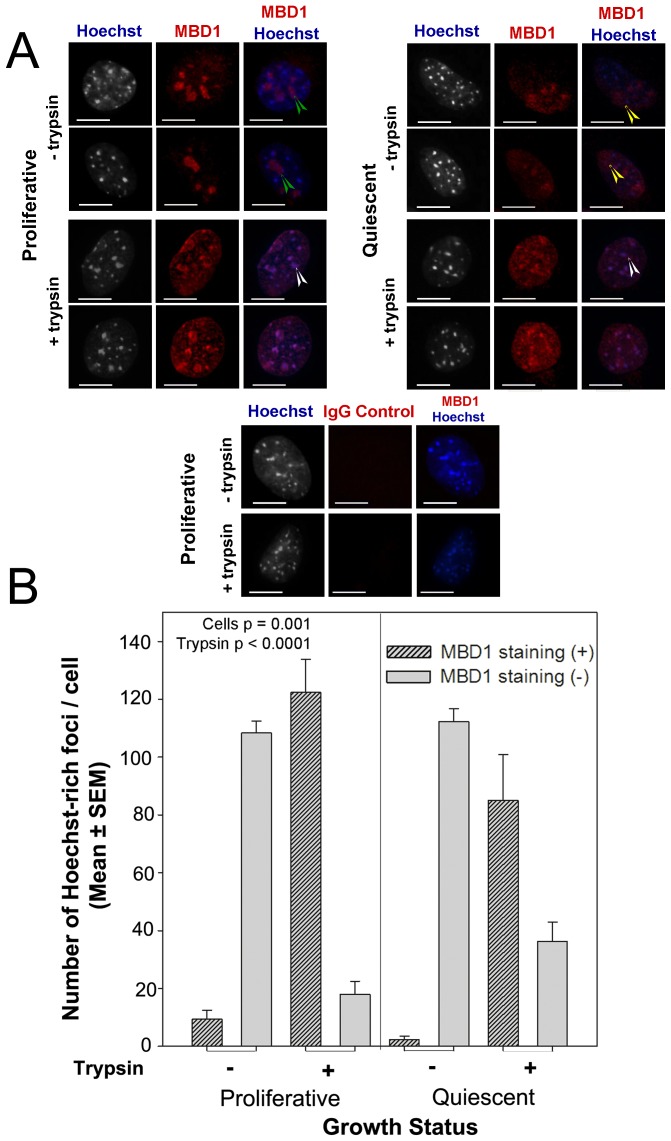
The effect of growth on the pattern of co-staining for MBD1 and bisBenzimide (Hoechst). **A** Co-staining of MBD1 (or non-immune IgG control) and DNA stain by Hoechst in proliferative and quiescent cells. Green, yellow and white arrowheads represent different localized MBD1 foci than Hoechst-rich foci. Scale bar 10 µm. **B** The number of Hoechst-dense regions showing coincident staining with MBD1 (+) or not (−). Cell growth (p = 0.001) affected this coincident staining and trypsin use (p<0.0001) affected this detection. The results are of three independent replicates.

A range of proteins, including MBD1, shows selective binding affinity for 5meC and their measurement is widely used as a proxy measure of 5meC. Immunolocalization of MBD1 showed different patterns of staining in proliferative and quiescent cells (**Fig. 6A**). Staining of MBD1 protein was evident after acid-only treatment (10 min) (**Fig. 6A**). In proliferative cells MBD1 staining appeared predominantly as a small number of large staining regions within nuclei. These generally did not coincide with the more numerous and smaller Hoechst-intense foci (green arrowheads, **Fig. 6A**). In quiescent cells MBD1 staining was more diffuse with some regionalised areas of more intense staining (yellow arrowheads, **Fig. 6A**), this staining did not predominantly accumulate within the Hoechst-intense foci (**Fig. 6A**). Tryptic digestion (1 min) resulted in an increased number of smaller MBD1 staining foci in both proliferative and quiescent cells. Some of this increased staining coincided with Hoechst-intense foci (white arrowheads, **Fig. 6A**). Numerical analysis showed that after tryptic-digestion the number of Hoechst-intense foci decorated by MBD1 was significantly increased (p<0.0001, **Fig. 6B**). The increased coincident staining was greater in proliferative cells (p≤0.001) (**Fig. 6B**). Thus, the MBD1 epitope showed two staining patterns within intact cells: a component that was solvent exposed after acid treatment which did not primarily associate with Hoechst-intense staining foci; and another pool that became solvent exposed only after trypsin treatment which tended to accumulate in regions coincident with Hoechst-intense foci.

**Figure 7:**
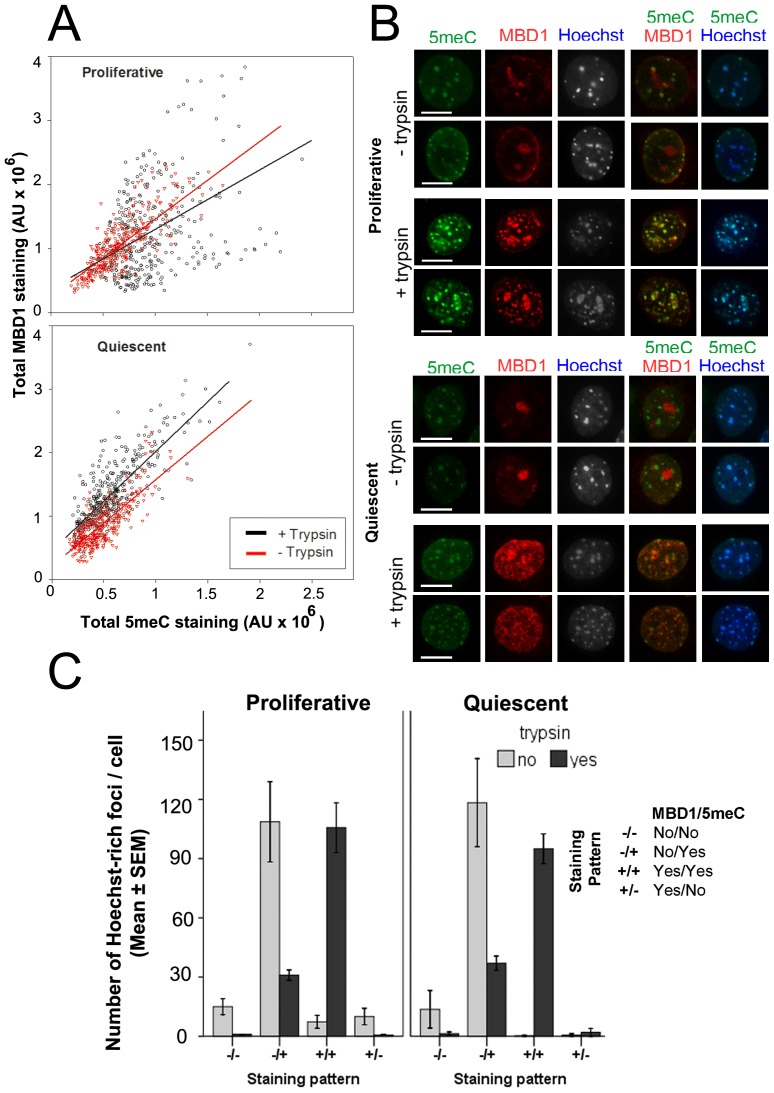
The effect of cell growth on nuclear staining patterns of 5meC and MBD1. **A** Proliferative and quiescent MEFs were treated with 10-stained for 5meC and MBD1. The positive association between the levels of 5meC and MBD1 staining was shown for each cell (AU = arbitrary units of optical density). **B** The localization of these antigens within the same nucleus. Scale bar 10 µm. **C** The number of Hoechst-intense regions of DNA with different staining defined as MBD1 (stained +, not-stained −) and 5meC (stained +, not-stained −). Those patterns are significantly different from each other (p<0.0001). The results are of three independent replicates.

Co-staining for MBD1 and 5meC showed that total staining levels of these two antigens was positively correlated in both proliferative and quiescent cells (**Fig. 7A**). In proliferative cells, trypsin treatment tended to reduce the correlation between staining of the two antigens (r^2^ = 0.55 without trypsin, r^2^ = 0.29 with trypsin), yet in quiescence cells the relationship was similar irrespective of tryptic digestion (r^2^ = 0.45 without trypsin, r^2^ = 0.41 with trypsin) (**Fig. 7A**). The apparent reduction in the coincident staining between the two antigen after trypsin treatment in proliferative cells was not significant (p>0.05) when tested by linear regression analysis due to the highly variable nature of the data. This result suggests it is likely that other factors contribute to the level of coincident 5meC and MBD1 staining levels in MEFs that were not captured within this model. Analysis of the distribution of staining within individual cells did, however, show some notable features. In the absence of tryptic digestion most of the Hoechst-intense foci displayed coincident 5meC staining but little detectable MBD1 (**Fig. 7B**). After tryptic digestion this pattern changed, with the majority of Hoechst-intense foci co-decorated with both anti-5meC and anti-MBD1. A significant minority of these Hoechst foci, however, remained decorated by anti-5meC but not MBD1 but it was relatively rare to observe Hoechst-intense foci that stained for MBD1 but not 5meC (**Fig. 7B, 7C**). The results indicate that MBD1 staining may not be an entirely reliable proxy measure for 5meC, particularly in the absence of tryptic digestion. Furthermore, there appears to be a significant pool of 5meC that was not co-decorated by MBD1.

## Discussion

This study redefines the conditions required for the reliable immunolocalization of the 5meC antigen within the fibroblast nucleus. It shows that conventional methods of acid-induced denaturation of chromatin allowed the solvent exposure of only a fraction of the total available 5meC antigen. Enhancing the antigen-retrieval process by an additional tryptic-digestion step revealed the presence of a substantial extra pool of antigen with fibroblasts. Use of this enhanced antigen retrieval process revealed that changed growth conditions of MEFs *in vitro* were associated with an increased total level of 5meC detected, and also showed that the relative distribution of the antigen between the acid-sensitive and trypsin-sensitive pools differed depending on the cell's growth state.

The proliferative status of the cell had the greatest effect on the observed total 5meC staining levels. MEFs in sparse culture in the exponential growth phase consistently showed higher 5meC staining levels than quiescent cells. Furthermore, cells that were aged *in vitro* (passage 7) had higher levels of staining than young cells. While these differences were evident after acid-induced denaturation alone, the dynamic range of the detected changes was greater after epitope retrieval by combined acid and trypsin treatment. A primary cause for this difference was the marked effect of the cell's growth status on the relative proportion of epitope detected within the trypsin-sensitive pool. In proliferative cells around half of the total staining was trypsin-sensitive but in quiescent cells (passage 1) this accounted for only around 25–30% of total staining. Immunolocalization is not a strictly quantitative tool, however, the study shows that the methodology can successfully detect the marked reduction in total methylation levels expected when proliferative cells are treated with an inhibitor of DNMT1 (which may be primarily responsible for the maintenance methylation of DNA during replication [19]). Thus, the methodology could successfully detect large relative changes in the net levels of 5meC in fibroblasts. The adaptation of the staining method to allow assessment by fluorescence-activated cytometry allowed large numbers of cells to be analysed and this improved its quantitative use.

The increase in 5meC levels detected in older cells contrasts with reported chemical analyses which generally indicate a small progressive loss of total cytosine modifications levels with increased time in culture [20]. It is noteworthy, however, that such analyses do not measure 5meC specifically but are a net measure of the range of known cytosine modifications [21]. More recent site-specific analyses of methylation changes suggest a more complex picture, whereby a large number of regions are differentially affected by either hypermethylation or hypomethylation as fibroblast age *in vitro* [22]. Hypermethylated regions were significantly over-represented in genes with developmental, immune-response and endocrine ontogenies [22]. The results and methods described here provide convenient tools for further analysis of the effect of the age and growth status of cells on changes in methylation levels.

The results show that the trypsin-sensitive masking of 5meC that was first discovered to occur during zygotic maturation [23], [24] can also occur to varying degrees in a somatic cell type. In the zygote, little trypsin-sensitive masking of 5meC occurred in chromatin immediately after fertilisation but a progressive increase occurred as zygote maturation progressed, so that by the time of syngamy most of the antigen was masked in a trypsin-sensitive manner. This period of zygotic maturation involved extensive remodelling of chromatin proteins and is associated with a generalised transcriptional repressive state [25]. It is also associated with reactivation of the mitotically active state from the mitotically quiescent state of the gametes. In the zygote, this masking did not appear to be primarily a fixation artefact since it was observed after either formaldehyde or methanol fixation of the cell [23]. Fibroblasts can also enter and leave the mitotic cycle *in vitro* and our experiments were designed to assess whether this phenomenon was associated with the masking observed in zygotes as they re-entered the mitotic cycle after fertilisation. We did not observe any growth conditions in MEFs that caused the almost complete trypsin-sensitive masking of 5meC that was observed in the zygote, yet it was clear that there was a relatively greater level of trypsin-sensitive masking in proliferative than in quiescent cells. While most cells in sparse culture are expected to be mitotically active, they will be at various stages of the cell-cycle. It is possible therefore that the variability in the 5meC measures detected represents cell-cycle associated differences but further analysis of this point is required. A more stable and substantial change to the cell's growth status is its exit from the cell-cycle into quiescence. Quiescent cells enter a special stage of the cycle known as G0. The reversible entry of fibroblast into G0 is a well characterised phenomenon [13]. It has a distinctive transcriptional profile, with overall changes in the level of transcription and chromatin organisation [13]. Entry into quiescence is generally associated with increased chromatin compaction [14] and it is therefore perhaps surprising that this was accompanied by reduced trypsin-sensitive masking of 5meC. However, this observation seems consistent with results of staining in the zygote where the level of 5meC epitope masking was greatest in the late zygote stage where chromatin structure is generally considered least compacted.

A major contributor to the extent of chromatin compaction is the formation of heterochromatin. A notable feature of the 5meC staining patterns observed in MEFs was that a proportion of the 5meC was detected within a large number of staining-intense foci scattered throughout the nucleus. Based upon their coincident staining with HP1β and also their enrichment for Hoechst stain, it is concluded that these were primarily heterochromatic foci. Tryptic digestion caused reduced staining of these regions with Hoechst and this was associated with an increase in 5meC staining, a result consistent with a role for the heterochromatic state of cells influencing the solvent exposure of 5meC. Methylation is known to be a major determinant of the recruitment of DNA to heterochromatin [26]–[29] so it was not surprising to find much of the cell's 5meC staining coincided with the heterochromatic foci within fibroblasts. It was more surprising to find that the level of solvent exposed 5meC detected within heterochromatin varied depending on the cell's growth state. Heterochromatin can account for up to a half of the genome [30], and it is considered to be a rather stable epigenetic feature within a given cell lineage. The similarity in Hoechst and HP1β staining in cells under each growth condition indicates that the overall level and distribution of heterochromatin in each of these conditions was similar. Yet, there is emerging body evidence indicating growth conditions can alter heterochromatin function. Heterochromatin is generally considered to result in the stable inactivation and stabilisation of repetitive DNA sequences and developmental regulatory genes that are not required to be expressed within a given lineage [30]. The accumulation of proteins of the HP1-family and histone modifying enzymes (such as Suv39h1α2 that catalyses the trimethylation of histone 3 at lysine 9) occur and these facilitate the recruitment of DNA methyltransferases [31], [32] resulting in the hypermethylation of heterochromatic regions. Together, these changes stabilise a transcriptionally repressive state, yet the extent of transcriptional repression within heterochromatin changes during the cell-cycle and as cells become quiescent. Transcription from pericentric heterochromatin increases during G1-phase, shows some repression in S-phase, but is not detected at all in quiescent cells [33]. There are also indications that cellular stress and also differentiation of cells can change the transcriptional activity within heterochromatin [34]. This plasticity of heterochromatin function suggests there may be considerable structural heterogeneity within heterochromatin depending upon the cell's growth and stress state. This study shows that conditions where transcriptional repression of heterochromatin is expected to be greatest (quiescent cells) is accompanied by overall lower levels of nuclear 5meC and reduced levels of trypsin-sensitive masking of the 5meC epitope. Further investigation is required to determine whether the level of solvent exposure of 5meC has a role in determining the transcriptional capacity of heterochromatin.

An important possible cause of antigenic masking of 5meC is the competition between endogenous binding proteins and the labelling antibodies for the antigen. Methylation of CpG dinucleotides provides a recognition and docking site for a range of 5meC-binding proteins. These contain an N-terminal consensus methyl binding domain (MBD), and include the MeCP2, MBD1 and MBD2 proteins, among several others. Although there is a high degree of conservation of the MBD domain within these proteins, other aspects of their structure allow them to show some selective binding to differing regions of the genome. There is also an emerging body of evidence of some DNA sequence selectivity in their binding [35]. These binding proteins are generally considered to serve as part of the transcriptional repressive environment created by CpG methylation [36]. This is achieved by their further recruitment of chromatin modifiers, for example, MBD1 proteins recruit and couple histone deacetylases and histone methylases [36], [37]. The combined effect is a more compact chromatin structure unfavourable to the formation of the transcriptional machinery at such sites.

The presence of high affinity methyl binding proteins might be a basis for the observed trypsin-sensitive masking of 5meC. Using MBD1 as an example of this class of proteins, we found that in acid denaturated cells much of the MBD1 staining was not coincident with 5meC, but this level of coincident staining increased after tryptic digestion. This result suggests MBD1 itself may also be the subject of antigen masking that is relieved by mild tryptic digestion. This further illustrates the complex antigenic environment created within the region of 5meC by changes in chromatin structure and conformation. It is likely that such changes account for dynamic and marked changes in the levels and nature of solvent exposure of the 5meC epitope. It also suggests that simple analyses of MBD1 (and presumably of members of the MBD1 family) should not be taken as a reliable proxy measure of global 5meC levels or localization without extensive validation. The persistence of MBD1 staining after tryptic digestion in the presence of enhanced 5meC staining might indicate that MBD1 is not a primary cause of the masking of 5meC. Yet, the anti-MBD1 used does not recognise the 5meC binding site so the possibility remains that changes in the level or affinity of methyl binding protein binding contributes to the masking.

The use of immunolocalization with specific antibodies allows the rapid assessment of changes in the relative levels of methylation and has the added advantage of allowing the detection of changes in the localization of methylation within the architecture of the nucleus [38], [39]. Chemical methods of analysing cytosine modifications at the base level require pooled DNA from a large number of cells and are therefore generally incompatible with the analysis of changes within individual cells. Immunolocalization has the further important advantage of being able to readily distinguish between the known range of modifications to cytosine (5meC, 5′-hydroxymethylcytosine, 5′-formylcytosine and 5′-carboxycytosine) within individual cells. Conventional methods of chemical analyses, such as bisulfite or Hpa2/Msp1 restriction analyses do not routinely achieve this discrimination [40], [41]. These advantages of immunolocalization of cytosine modifications mean it is an important tool for epigenetic research, but its meaningful use requires that the conditions required for valid detection of 5meC *in situ* are understood and defined.

This study shows that immuno-detectable 5meC exists in two pools within MEFs; being acid sensitive and trypsin-sensitive pools. These two pools were not stable, however, with conditions such as the cell's growth and age in culture affecting the distribution of 5meC between the two. Proliferative cells had higher overall levels of 5meC staining and a much greater proportion of this 5meC within the trypsin-sensitive pool then for cells that had withdrawn from the cell-cycle. This finding extends an earlier observation that 5meC in the newly fertilised zygotes was mainly within the acid-sensitive pool but as zygote maturation proceeded there was a progressive and almost complete conversion of antigens from the acid-sensitive to the trypsin-sensitive pool. These new observations show that antigenic presentation of 5meC is a highly dynamic epigenetic feature and is likely to be subject of changes induced by the dynamic nature of chromatic structure and conformation. This work provides tools required for the reliable immunological detection of 5meC within fibroblasts and will allow assessment of the functional significance of changes in the conditions of solvent exposure of 5meC with the cell's growth status.

## Materials and Methods

### Cell culture

Mouse embryonic fibroblasts (MEFs) from Day 13.5 embryos were collected using standard methods and then cultured in Dulbecco's modified eagle medium (DMEM) (Thermo Fisher Scientific Inc, Utah, USA; Cat. No. SH30243.FS). Media were supplemented with 10% (v/v) fetal bovine serum (FBS) (Invitrogen Life Technologies, Carlsbad, USA; Cat. No. 10099-141), 1% (v/v) 1× MEM-Non-Essential Amino Acids solution (Invitrogen, Cat. No. 11140-050), 50 U/ml penicillin (Sigma-Aldrich Co.; St. Louis, MO, USA; Cat. No P3032) and 50 µg/ml streptomycin (Sigma, Cat. No. S6501) at 37°C with 5% CO_2_ in air. Cells were grown as proliferative (1 day-culture), confluent (3-day culture) or quiescent (24 h serum-starved confluent culture) cultures. Cells were passages 0–7. This study was carried out in strict accordance with the Australian Code of Practice for the Care and Use of Animals for Scientific Purposes. The protocol was specifically approved by the Royal North Shore Hospital Animal Care and Ethics Committee (Protocol number 0802-003). No procedures were performed on living animals.

### Immunolocalization of MEFs

MEFs were washed with Ca^2+^, Mg^2+^ free Dulbecco's phosphate buffered saline (DPBS) (Invitrogen, Cat No. 14190-144) at 37°C and fixed with 4% (w/v) fresh paraformaldehyde (PFA) (Sigma, Cat. No. P6148) for 30 min at 22°C. Cells were permeabilized with phosphate buffered saline (Sigma, Cat No. D5773) containing 0.75% (v/v) Tween-20 (Sigma, Cat. No. P7949) and 0.75% (v/v) Triton-×100 (Bio-Rad Laboratories In., CA, USA, Cat. No. 161-0407) in PBT (phosphate-buffered saline with tween-20) for 1 h at RT. Antigen retrieval procedures were performed as defined in each experiment. We used two approaches; one involved denaturation of chromatin by brief exposure to acid conditions, and the other approach was to follow the acid-induced denaturation [42] by an additional step of brief tryptic digestion [11]. Following fixation and permeabilization chromatin was denaturated by treatment with HCl (4N) treatment either for 10 or 20 min at 22°C. Cells were washed with PBT for 30 min. This was followed by brief digestion with trypsin (0.25%) (Invitrogen, Cat. No. 25200-56) for various times (0, 1 or 2 min as shown in each relevant experiments) at 37°C (Trypsin treatment was not applied for HP1-β staining). Trypsin was inactivated by addition of equal volume of pre-warmed media DMEM containing 3 mg/ml BSA (Sigma, Cat.No. A1470) and 10% (v/v) sheep serum (Sigma, Cat.No. S3772) for 2 min at 37°C and washed with PBT. They were then blocked in 30% (v/v) sheep serum in PBT at 4°C overnight. Cells were incubated in primary antibodies followed by staining with secondary antibodies conjugated with an appropriate fluorophore. In all experiments a negative control was included in the design of incubation with non-immune immunoglobulin and secondary antibodies.

The primary antibodies used in the study were diluted in PBT with 2 mg/ml BSA: mouse monoclonal anti-5meC (1∶60), (Clone 33D3, AbD Serotec, Raleigh, NC) for 1 h at RT; rabbit polyclonal antibody against MBD1 (1∶50), (Abcam, UK, Cat. No. ab3753) at 4°C overnight; mouse monoclonal anti-HP1-β (1∶200) (Abcam, Cat. No. Ab101425) at 4°C overnight. Where indicated, cells were counter-stained for DNA with 4 µg/ml Hoechst 33342 (Sigma, Cat. No. B2261).

Double staining for 5meC and MBD1 was performed as follows. Cells were incubated in rabbit anti-MBD1 (1∶50) or non-immune rabbit IgG (1∶50) (Sigma, Cat. No. I5006) overnight at 4°C. Cells were washed with PBT for 10 min at RT followed by incubation with mouse anti-5meC (1∶60) or non-immune mouse IgG (1∶60) (Sigma, Cat. No. M7894) in 2 mg/ml BSA in PBT for 1 h at RT. Cells were incubated with secondary antibody mix including anti rabbit-Texas Red (1∶200) (Abcam, Cat. No. ab6719) and anti mouse-FITC (1∶200) (Sigma, Cat. No. 6257) for 1 h in the dark at RT and counter-stained with Hoechst (4 µg/ml) (Sigma, Cat. No. B2261).

### Inhibition of DNMT

Methylation of cells during their replication was blocked by treatment with 5-Aza-2′-deoxycytidine (5-AZA) (Sigma, Cat No. A3656) at concentrations of 0 and 5 µM in culture media for 24 h.

### Fluorescence activated flow cytometric analysis of 5meC staining

After treatment, cells were washed with 1×PBS and detached by 0.25% trypsin- EDTA for up to 5 min. Trypsin was inactivated with an equal volume of DMEM/FBS (complete) media and removed by 4 min centrifugation at 400 g. Cells were washed with 1×PBS (w/v) and fixed with 4% (w/v) PFA for 30 min. Fixed cells were permeabilized by 1×PBT (1×PBS including 0.75% (v/v) tween-20 and 0.75% (v/v) triton-X) for 1 h. Chromatin was denaturated by treatment with 4N HCl for 10 min. Cells were washed with 1×PBS for 30 min to remove acid and treated with trypsin (for 1 min) after acid. Following antigenic retrieval, cells were blocked in 30% (v/v) sheep serum (Sigma, Cat. No. S3772) in 1×PBT overnight. Cells were stained in primary antibodies (1∶60) mouse monoclonal anti-5-methylcytosine (AbD Serotec Ltd, UK; Cat. No 5MCA2201) or a non-immune mouse IgG (Sigma; Cat. No. M7894) in 2 mg/ml BSA in 1×PBT for 1 h, and then washed with 1×PBS for 30 min. Cells then were incubated with a PerCP-conjugated secondary antibody (1∶21) (R&D Systems, MN, USA;, Cat. No. F0114) (in 1×PBS) for 30 min in the dark. Cells were washed in 1×PBS 3 times by centrifugation at 900 g for 5 min. All steps, excluding blocking and PerCP incubation at 4°C were performed at room temperature. Stained cells were analysed by flow cytometry. At least 10,000 total events were acquired for each treatment per replicate. Total 5meC methylation was gathered at fluorescence channel-3 (FL3-H) for PerCP label (excitation at 488 nm, emission at 678 nm). Flow cytometry instrument (BD FACSCalibr, BDIS San Jose Ca. USA) was set for maximum fluorochrome sensitivity using CalibrateTM standardisation microspheres.

### Microscopy and image analysis

Cells were then assessed by epifluorescence microscopy, Eclipse 80i (Nikon Instruments Inc., USA). Epifluorescence microscopy images were captured using CoolSnap cf camera (Photometrics, AZ., USA) and total 5meC staining in nuclei measured using Image-Pro Plus version 5.0 (Media Cybernetics, Inc., MD). Slides were assessed using the same setting of UV power, and images were taken using the same exposure time (4 sec) for all treatments of replicates in each experiment. Original images were without analysed any further adjustments. Merged versions of images were in original colours and optimisation of contrast and color intensity was applied to all treatments and images. Nuclei were randomly selected using drawing tool of Image-Pro and analysed. The total amount of nuclear staining of specific antigens was analysed for at least 80–100 nuclei in each treatment per replicate. The total staining (sum value) of specific antigens in each cell (nucleus) was measured, and the level of staining is optical density (arbitrary units). The average staining of antigen in cells was shown using bar graphs. The average number of nuclear foci per cell (nucleus) was shown in bar graphs.

### Statistics

Statistical analyses were performed using SPSS software, Version 19.0. Total amount of 5meC staining and the number of 5meC foci and their sizes within nuclei were compared by univariate analysis using the general linear method. In each case the replicate was incorporated into the model as a covariate. The distribution of 5meC staining (focal or diffuse) was compared by binominal logistic regression analysis. The relationship between 5meC and MBD1 was analysed by linear regression. Analyses include data from at least 3 independent replicates of each treatment per experiment.

## References

[1] Hajkova P (2010) Epigenetic reprogramming - taking a lesson from the embryo. Current Opinion in Cell Biology 22: 342–350. 20537882 10.1016/j.ceb.2010.04.011

[2] Momparler R, Bovenzi V (2000) DNA Methylation and Cancer. Journal of Cellular Physiology 183: 145–154. 10737890 10.1002/(SICI)1097-4652(200005)183:2<145::AID-JCP1>3.0.CO;2-V

[3] Ooi SKT, O'Donnell AH, Bestor TH (2009) Mammalian cytosine methylation at a glance. Journal of Cell Science 122: 2787–2791. 19657014 10.1242/jcs.015123 PMC2724605

[4] Morgan H, Santos F, Green K, Dean W, Reik W (2005) Epigenetic Reprogramming in Mammals. Hum Mol Genet 14: 47–58. 10.1093/hmg/ddi114 15809273

[5] Oswald J, Engemann S, Lane N, Mayer W, Olek A, et al (2000) Active demethylation of the paternal genome in the mouse zygote. Current Biology 10: 475–478. 10801417 10.1016/s0960-9822(00)00448-6

[6] Morgan HD, Santos F, Green K, Dean W, Reik W (2005) Epigenetic reprogramming in mammals. Human Molecular Genetics 14: R47–58. 15809273 10.1093/hmg/ddi114

[7] Wossidlo M, Arand J, Sebastiano V, Lepikhov K, Boiani M, et al (2010) Dynamic link of DNA demethylation, DNA strand breaks and repair in mouse zygotes. EMBO J 29: 1877–1888. 20442707 10.1038/emboj.2010.80 PMC2885932

[8] Smith ZD, Chan MM, Mikkelsen TS, Gu H, Gnirke A, et al (2012) A unique regulatory phase of DNA methylation in the early mammalian embryo. Nature 484: 339–344. 22456710 10.1038/nature10960 PMC3331945

[9] Smallwood SA, Tomizawa S, Krueger F, Ruf N, Carli N, et al (2011) Dynamic CpG island methylation landscape in oocytes and preimplantation embryos. Nature Genetics 43: 811–U126. 21706000 10.1038/ng.864 PMC3146050

[10] Mayer W, Niveleau A, Walter J, Fundele R, Haaf T (2000) Demethylation of the zygotic paternal genome. Nature 403: 501–502. 10676950 10.1038/35000656

[11] Li Y, O'Neill C (2012) Persistance of Cytosine Methylation of DNA following Fertilisation in the Mouse. PloS One 7. 10.1371/journal.pone.0030687 PMC3266909 22292019

[12] Li Y, O'Neill O (2013) 5′-methylcytosine and 5′-hydroxymethylcytosine Each Provide Epigenetic Information to the Mouse Zygote. PloS ONE 8: e63689. 23691085 10.1371/journal.pone.0063689 PMC3653832

[13] Coller HA, Sang L, Roberts JM (2006) A New Description of Cellular Quiescence. PLoS Biol 4: e83. 16509772 10.1371/journal.pbio.0040083 PMC1393757

[14] Evertts AG, Manning AL, Wang X, Dyson NJ, Garcia BA, et al (2013) H4K20 methylation regulates quiescence and chromatin compaction. Molecular Biology of the Cell 24: 3025–3037. 23924899 10.1091/mbc.E12-07-0529 PMC3784377

[15] Jones PA, Taylor SM (1980) Cellular differentiation, cytidine analogs and DNA methylation. Cell 20: 85–93. 6156004 10.1016/0092-8674(80)90237-8

[16] Grigoryev SA, Nikitina T, Pehrson JR, Singh PB, Wodcock CL (2004) Dynamic relocation of epigenetic chromatin markers reveals an active role of constitutive heterochromatin in the transition from proliferation and quiescence. Journal of Cell Science 117: 6153–6162. 15564378 10.1242/jcs.01537

[17] Festenstein R, Pagakis SN, Hiragami K, Lyon D, Verreault A, et al (2003) Modulation of heterochromatin protein 1 dynamics in primary mammalian cells. Science 299: 719–721. 12560554 10.1126/science.1078694

[18] Lomberk G, Wallrath LL, Urrutia R (2006) The Heterochromatin Protein 1 family. Genome Biology 7. 10.1186/gb-2006-7-7-228 PMC1779566 17224041

[19] Bestor TH (2000) The DNA methyltransferases of mammals. Human Molecular Genetics 9: 2395–2402. 11005794 10.1093/hmg/9.16.2395

[20] Wilson VL, PA J (1983) DNA methylation decreases in aging but not in immortal cells. Science 220: 31055–31057. 10.1126/science.6844925 6844925

[21] Huang Y, Pastor WA, Shen Y, Tahiliani M, Liu DR, et al (2010) The Behaviour of 5-Hydroxymethylcytosine in Bisulfite Sequencing. PLoS ONE 5: e8888. 20126651 10.1371/journal.pone.0008888 PMC2811190

[22] Koch CM, Suschek CV, Lin Q, Bork S, Goergens M, et al (2011) Specific Age-Associated DNA Methylation Changes in Human Dermal Fibroblasts. PLoS ONE 6: e16679. 21347436 10.1371/journal.pone.0016679 PMC3035656

[23] Li Y, O'Neill C (2012) Persistence of Cytosine Methylation of DNA following Fertilisation in the Mouse. PLoS ONE 7: e30687. 22292019 10.1371/journal.pone.0030687 PMC3266909

[24] Li Y, O'Neill C (2013) 5′-methylcytosine and 5′-hydroxymethylcytosine Each Provide Epigenetic Information to the Mouse Zygote. PLoS ONE 8: e63689. 23691085 10.1371/journal.pone.0063689 PMC3653832

[25] Latham KE, Solter D, Schultz RM (1992) Acquisition of a transcriptionally permissive state during the 1-cell stage of mouse embryogenesis. Developmental Biology 149: 457–462. 1309712 10.1016/0012-1606(92)90300-6

[26] Akhmanova A, Verkerk T, Langeveld A, Grosveld F, Galjart N (2000) Characterisation of transcriptionally active and inactive chromatin domains in neurons. Journal of Cell Science 113: 4463–4474. 11082040 10.1242/jcs.113.24.4463

[27] Hajkova P, Ancelin K, Waldmann T, Lacoste N, Lange U, et al (2008) Chromatin Dynamics during Epigenetic Reprogramming in the Mouse Germ Line. Nature 452: 877–881. 18354397 10.1038/nature06714 PMC3847605

[28] Kang YK, Koo DB, Park JS, Choi YH, Lee KK, et al (2001) Differential inheritance modes of DNA methylation between euchromatic and heterochromatic DNA sequences in ageing fetal bovine fibroblasts. FEBS Letters 498: 1–5. 11389887 10.1016/s0014-5793(01)02472-3

[29] Wongtawan T, Taylor JE, Lawson KA, Wilmut I, Pennings S (2011) Histone H4K20me3 and HP1 alpha are late heterochromatin markers in development, but present in undifferentiated embryonic stem cells. Journal of Cell Science 124: 1878–1890. 21576353 10.1242/jcs.080721

[30] Grewal SIS, Moazed D (2003) Heterochromatin and Epigenetic Control of Gene Expression. Science 301: 798–802. 12907790 10.1126/science.1086887

[31] Probst AV, Almouzni G (2011) Heterochromatin establishment in the context of genome-wide epigenetic reprogramming. Trends in Genetics 27: 177–185. 21497937 10.1016/j.tig.2011.02.002

[32] Probst AV, Dunleavy E, Almouzni G (2009) Epigenetic inheritance during the cell cycle. Nat Rev Mol Cell Biol 10: 192–206. 19234478 10.1038/nrm2640

[33] Lu J, Gilbert DM (2007) Proliferation-dependent and cell cycle–regulated transcription of mouse pericentric heterochromatin. The Journal of Cell Biology 179: 411–421. 17984319 10.1083/jcb.200706176 PMC2064789

[34] Chan FL, Wong LH (2012) Transcription in the maintenance of centromere chromatin identity. Nucleic Acids Research 40: 11178–11188. 23066104 10.1093/nar/gks921 PMC3526279

[35] Clouaire T, de las Heras JI, Merusi C, Stancheva I (2010) Recruitment of MBD1 to target genes requires sequence-specific interaction of the MBD domain with methylated DNA. Nucleic Acids Research 38: 4620–4634. 20378711 10.1093/nar/gkq228 PMC2919722

[36] Rountree MR, Bachman KE, Herman JG, Baylin SB (2001) DNA methylation, chromatin inheritance, and cancer. Oncogene 20: 3156–3165. 11420732 10.1038/sj.onc.1204339

[37] Sarraf SA, Stancheva I (2004) Methyl-CpG Binding Protein MBD1 Couples Histone H3 Methylation at Lysine 9 by SETDB1 to DNA Replication and Chromatin Assembly. Molecular Cell 15: 595–605. 15327775 10.1016/j.molcel.2004.06.043

[38] Brown SE, Weaver ICG, Meaney MJ, Szyf M (2008) Regional-specific global cytosine methylation and DNA methyltransferase expression in the adult rat hippocampus. Neuroscience Letters 440: 49–53. 18539393 10.1016/j.neulet.2008.05.028

[39] Miller OJ, Schnedl W, Allen J, Erlanger BF (1974) 5-Methylcytosine Localized in Mammalian Constitutive Heterochromatin. Nature 251: 636–637. 4609195 10.1038/251636a0

[40] Huang Y, Pastor WA, Shen YH, Tahiliani M, Liu DR, et al (2010) The Behaviour of 5-Hydroxymethylcytosine in Bisulfite Sequencing. Plos One 5. 10.1371/journal.pone.0008888 PMC2811190 20126651

[41] Yu M, Hon G, Szulwach K, Song C, Zhang L, et al (2012) Base-Resolution Analysis of 5-Hydroxymethylcytosine in the Mammalian Genome. Cell 149: 1368–1380. 22608086 10.1016/j.cell.2012.04.027 PMC3589129

[42] Santos F, Hendrich B, Reik W, Dean W (2002) Dynamic Reprogramming of DNA Methylation in the Early Mouse Embryo. Developmental Biology 241: 172–182. 11784103 10.1006/dbio.2001.0501

